# Orbitadekompression bei endokriner Orbitopathie – Erfahrungen und Ergebnisse

**DOI:** 10.1007/s00347-020-01181-8

**Published:** 2020-07-17

**Authors:** Sebastian Küchlin, Markus Gruber, Michael Reich, Lutz Joachimsen, Marc Metzger, Jürgen Beck, Jürgen Grauvogel, Wolf A. Lagrèze

**Affiliations:** 1 Klinik für Augenheilkunde, Universitätsklinikum Freiburg, Killianstr. 5, 79106 Freiburg, Deutschland; 2 Interdisziplinäres Orbitazentrum, Universitätsklinikum Freiburg, Freiburg, Deutschland; 3 Medizinische Fakultät, Albert-Ludwigs-Universität Freiburg, Freiburg, Deutschland; 4 Klinik für Mund‑, Kiefer- und Gesichtschirurgie, Universitätsklinikum Freiburg, Freiburg, Deutschland; 5 Klinik für Neurochirurgie, Universitätsklinikum Freiburg, Freiburg, Deutschland

**Keywords:** Laterale Dekompression, Pterionale Dekompression, Morbus Basedow, Exophthalmus, Orbita, Deep lateral wall resection, Pterional decompression, Graves’ Disease, Exophthalmos, Orbit

## Abstract

**Hintergrund:**

Die endokrine Orbitopathie ist die häufigste extrathyreoidale Manifestation des Morbus Basedow und tritt bei schätzungsweise 25–50 % der betroffenen Patienten auf. Krankheitsbedingt kommt es zu einer entzündlichen Schwellung der Orbitaweichteile. Die Behandlung erfolgt meist konservativ. Bei schweren Fällen mit beeinträchtigendem Exophthalmus oder akuter, steroidrefraktärer Visusbedrohung kann eine chirurgische Orbitadekompression die Beschwerden der Patienten lindern oder das Sehvermögen erhalten. Ein wesentlicher Aspekt der Versorgungsqualität besteht in der Vermeidung postoperativer Doppelbilder.

**Ziel der Arbeit:**

Erfahrungs- und Ergebnisbericht von 100 chirurgischen Orbitadekompressionen bei 62 Patienten an einem interdisziplinären Orbitazentrum. Patienten mit Kompression der Orbitaspitze wurden mittels pterionaler Dekompression behandelt. Patienten ohne Hinweise auf Orbitaspitzenbeteiligung wurden mittels tiefer lateraler Wandresektion oder pterionaler Dekompression behandelt.

**Methodik:**

Retrospektive Datenanalyse.

**Ergebnisse:**

Die mittlere Exophthalmusreduktion betrug 2,9 mm. Augen mit visusbedrohendem Schweregrad gewannen im Mittel 2,2 Zeilen an Sehschärfe, der Visus bei rehabilitativer Indikation blieb stabil. Die Komplikationsrate betrug 4 %. Neue Doppelbilder wurden nach 2 Eingriffen beobachtet. Bei einem Patienten kam es zu einer Visusminderung von 0,8 auf 0,1. In 9 Fällen führte die Operation zu einem vollständigen Rückgang zuvor beklagter Doppelbilder.

**Diskussion:**

Visusgewinn, Exophthalmusreduktion und Komplikationsrate sind in diesem Kollektiv vergleichbar mit zuvor publizierten Arbeiten. Diese Studie bestätigt die Rolle der Orbitadekompression bei visusbedrohender und schwer beeinträchtigender endokriner Orbitopathie.

## Abstract (en)

**Background:**

Graves’ orbitopathy is the most frequent extrathyroidal manifestation of Gravesʼ disease, affecting approximately 25–50% of patients. It leads to inflammation and swelling of orbital soft tissues. The treatment is mostly conservative. Surgical orbital decompression is indicated in severe cases with disfiguring exophthalmos or an acute steroid-refractive threat to vision, facilitating visual and cosmetic recovery. An important aspect in the quality of care is the avoidance of postoperative diplopia.

**Objective:**

To report experiences and results from 100 cases of orbital decompression surgery performed on 62 patients at a multidisciplinary orbit center. Patients with signs of apical crowding were treated by pterional decompression. Patients without signs of apical crowding were treated either by deep lateral wall resection or pterional decompression.

**Methods:**

A retrospective data analysis was carried out.

**Results:**

The mean reduction in exophthalmos was 2.9 mm. Visual acuity improved by a mean of 2.2 lines in eyes with sight-threatening disease. In moderate to severe disease, visual acuity remained stable. The complication rate was 4%. New postoperative diplopia occurred after two interventions and one patient experienced a deterioration in visual acuity from 0.8 to 0.1. In nine cases, surgery led to a complete regression of previously reported double vision.

**Conclusion:**

Visual acuity gain, reduction of exophthalmos and complications in this collective are comparable to previously published results. The results of this study confirm the role of orbital decompression in the treatment of sight-threatening and severely disfiguring endocrine orbitopathy.

Die endokrine Orbitopathie (EO) ist die häufigste extrathyreoidale Manifestation des Morbus Basedow und tritt bei schätzungsweise 25–50 % der betroffenen Patienten auf. Krankheitsbedingt kommt es zu einer entzündlichen Schwellung der Orbitaweichteile. Die Behandlung erfolgt meist konservativ. In schweren Fällen mit entstellendem Exophthalmus oder akuter Visusbedrohung kann eine chirurgische Orbitadekompression die Beschwerden der Patienten lindern oder das Sehvermögen erhalten. Ein wesentlicher Aspekt der Versorgungsqualität besteht in der Vermeidung postoperativer Doppelbilder. Aktuell zeigt sich ein Trend zur befund- und patientenorientierten Wahl des chirurgischen Verfahrens. In diesem Beitrag berichten wir von unseren Erfahrungen mit der chirurgischen Dekompression von 100 Orbitae an 62 Patienten mittels zweier Verfahren im Zeitraum 2000 bis 2019.

Die endokrine Orbitopathie (EO) ist eine häufige extrathyreoidale Manifestation von autoimmunen Schilddrüsenerkrankungen, insbesondere des Morbus Basedow. Bedingt durch eine entzündliche Volumenzunahme des intraorbitalen Muskel- und Fettgewebes, kann es zu Exophthalmus, Motilitätseinschränkungen, Doppelbildern, Fremdkörpergefühl und Hornhautstippung kommen. In schweren Fällen ist das Sehvermögen durch Expositionskeratopathie oder einen kompressionsbedingten Sehnervschaden (dysthyreote Optikusneuropathie) bedroht [1].

## Die Behandlung der endokrinen Orbitopathie richtet sich nach Krankheitsaktivität und -schweregrad

Der typische Verlauf der Erkrankung besteht aus einer initialen Phase hoher Aktivität, welche nach ca. 18 bis 24 Monaten in ein inaktives, fibrotisches Stadium übergeht [2]. Eine Objektivierung der klinischen Aktivität erfolgt durch den *Clinical Activity Score* (CAS) [3]. Die *European Group on Grave’s Orbitopathy* (EUGOGO) unterscheidet hier zwischen einer „aktiven“ und „nicht aktiven“ Erkrankung. Ein CAS ≤3 wird dabei oft als inaktive Krankheit definiert, ab einem CAS von ≥4 spricht man von einem aktiven Krankheitsstadium [4]. Der Schweregrad der EO wird durch einen klinischen Score („NOSPECS^1^“) auf einer Skala von 0 bis 16 Punkten bestimmt [6]. Ergänzend hierzu unterscheidet man nach der EUGOGO-Klassifikation 3 Grade: „mild“, „moderat bis schwer“ und „visusbedrohend“ [4].

Die Versorgung der Patienten orientiert sich an den zuvor genannten Parametern und erfolgt überwiegend konservativ. Der Konsensus der EUGOGO empfiehlt, alle Patienten mit einer Lokaltherapie (z. B. Tränenersatzmittel) zu behandeln und nach Möglichkeit in einem spezialisierten Zentrum anzubinden [7]. Wichtig ist die optimale internistische Einstellung der Schilddrüsenfunktion. Prummel et al. zeigten 1993, dass die EO bei Rauchern häufiger auftritt als bei Nichtrauchern und einen schwereren Verlauf nimmt [8]. Patienten, bei denen ein Nikotinkonsum vorliegt, sollen daher unbedingt zu einem Rauchstopp ermuntert werden [4]. Patienten mit „milder“ Krankheitsausprägung können mit Selen therapiert werden, was den Krankheitsverlauf abmildert und die Lebensqualität verbessert [9]. Eine „moderat bis schwer“ ausgeprägte EO im aktiven Stadium rechtfertigt eine Behandlung mit i.v.-Glukokortikoiden, üblicherweise mit 500 mg Methylprednisolon wöchentlich für 6 Wochen, dann 250 mg wöchentlich für weitere 6 Wochen („Mainzer Schema“) [10]. Liegt eine „visusbedrohende“ Orbitopathie mit Kompression des N. opticus vor, ist eine i.v.-Therapie mit einer Megadosis Methylprednisolon indiziert (1000 mg/Tag für 3 Tage) [7, 11]. Bringt diese keine ausreichende Besserung, ist eine baldige Druckentlastung durch eine chirurgische Orbitadekompression angezeigt. Eine Dekompression kann ebenfalls im „moderat bis schweren“ Stadium erwogen werden, sofern das Krankheitsgeschehen inaktiv ist und eine subjektiv schwere Beeinträchtigung durch stigmatisierenden Exophthalmus vorliegt [7].

## Limitierte Datenlage trotz hoher Prävalenz

Obwohl die OD fester Bestandteil des Behandlungsschemas der EO ist, besteht diesbezüglich weiterhin ein Mangel an hochqualitativen Daten. Es wurde eine Vielzahl unterschiedlicher Zugänge und Operationstechniken von Operateuren aus den Fachbereichen Augenheilkunde, HNO, Mund‑, Kiefer- und Gesichtschirurgie, Neurochirurgie und plastisch-rekonstruktive Chirurgie entwickelt. Die Vergleichbarkeit der Eingriffe ist durch verschiedene Methodik und unterschiedliche Patientenkollektive eingeschränkt [12]. Stetige Weiterentwicklungen der Operationstechniken haben die Komplikationsrate orbitachirurgischer Eingriffe gesenkt [13, 14]. Ein wichtiger Aspekt hierbei ist die Vermeidung postoperativer Doppelbilder, welche bei früheren Dekompressionstechniken in bis zu zwei Dritteln aller Fälle auftraten [15]. Neben anderen Verfahren haben sich die (tiefe) laterale Dekompression und die pterionale Dekompression diesbezüglich als günstig erwiesen [16, 17]. Ein weiterer Ansatz zur Optimierung der Versorgungsqualität besteht in der patientenzentrierten Wahl des Operationsverfahrens [18].

Ziel der vorliegenden Arbeit ist es, von unseren Erfahrungen mit 2 Operationstechniken der OD zu berichten, die durch die Ophthalmo- und MKG-Chirurgie sowie die Neurochirurgie an einem interdisziplinären Orbitazentrum durchgeführt wurden. Dabei legen wir ein besonderes Augenmerk auf eine umfassende Charakterisierung unseres Patientenkollektivs, um eine größtmögliche Vergleichbarkeit zu gewährleisten.

## Patienten und Methodik

Bei dieser Arbeit handelt es sich um eine retrospektive Datenanalyse. Eingeschlossen wurden alle Patienten, welche im Zeitraum vom 01.01.2000 bis 31.06.2019 an der Klinik für Augenheilkunde der Universitätsklinik Freiburg vorstellig wurden und im Verlauf eine knöcherne Orbitadekompression erhielten (*n* = 62 Patienten, *n* = 100 operierte Orbitae). Ausgeschlossen wurden Patienten mit Vorgeschichte einer Orbitadekompression am betroffenen Auge, Patienten, zu denen unzureichende Daten zur Verfügung standen, sowie Patienten, bei denen eine Dekompression von Orbitafettgewebe ohne knöcherne Dekompression durchgeführt wurde. Zwei eingeschlossene Patienten erhielten im Verlauf eine Re-Dekompression einer Orbita, hier wurde jeweils nur der erste Eingriff für die Analyse verwertet. Es erfolgte eine systematische Erfassung der Patientenvorgeschichte, klinischer Parameter sowie aller dokumentierter Komplikationen als Resultat der medizinischen und chirurgischen Behandlung. Als wichtigste Endpunkte wurden die Angabe von Doppelbildern im Gebrauchsblickfeld (Geradeausblick ±20°) vor und nach der Operation, der prä- und postoperative bestkorrigierte Visus und der prä- und postoperative Exophthalmus (Hertel-Exophthalmometer) am operierten Auge und am Partnerauge erfasst. Patienten, bei denen ein CAS ≥4 erhoben war, wurden nach EUGOGO als „aktiv“ klassifiziert. Bei Patienten, die unmittelbar vor dem Eingriff eine i.v.-Steroidtherapie erhielten, wurden, wenn verfügbar, beide präoperativen Parameter nach Abschluss der Steroidtherapie und vor dem operativen Eingriff erhoben. Als postoperativer Wert wurde ein repräsentativer Wert aus den verfügbaren Nachuntersuchungen gewählt. In allen Fällen waren die postoperativen Werte vor einer etwaigen operativen Versorgung des FE erhoben worden. Unterschiede zwischen prä- und postoperativen Werten wurden als Mittelwert der individuellen Differenzen berechnet. In einem Subkollektiv analysierten wir das erste operierte Auge im Vergleich mit dem nicht operierten Partnerauge (*n* = 62 Patienten, *n* = 62 Orbitae). Die Signifikanz der Ergebnisse wurde mit dem t‑Test nach Student geprüft. Patientenspezifische Effekte wurden durch ein lineares *multilevel model* mit *random intercepts* pro Patient modelliert und die Ergebnisse mit dem t‑Test verglichen. Die statistische Datenanalyse erfolgte in R (Version 3.6.2), RStudio (RStudio, PBC, Boston, USA) (Version 1.0.143) und dem Paket nlme [19] (Version 3.1-144). Die grafische Aufarbeitung der Ergebnisse erfolgte in R mit dem Paket ggplot2 (Version 3.2.1).

## Behandlungsschemata und Wahl der Operationstechnik

**Figure 1:**
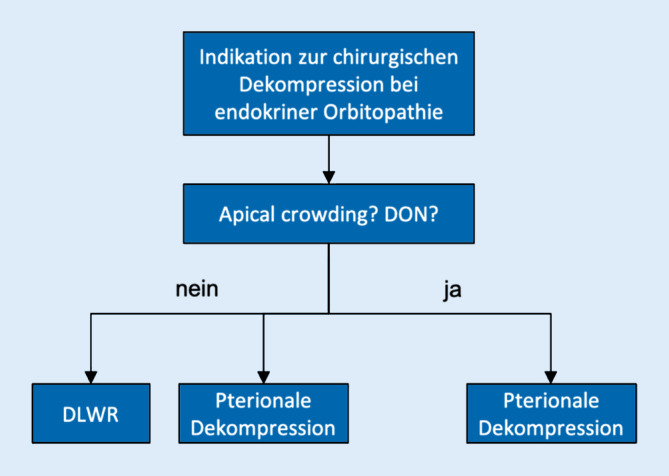

Im Falle eines subjektiv schwer beeinträchtigenden Exophthalmus erfolgte nach sorgfältiger Indikationsstellung und Sicherstellung eines klinisch inaktiven Krankheitsstadiums eine operative Entlastung über einen anterioren Zugang durch einen Operateur der Klinik für Augenheilkunde und MKG-Chirurgie. Patienten mit dysthyreoter Optikusneuropathie wurden notfallmäßig stationär aufgenommen und mit 1000 mg Methylprednisolon i.v. für 3 Tage behandelt. Alle Patienten wurden in unserer interdisziplinären Schädelbasiskonferenz vorgestellt. Patienten mit rehabilitativer Indikation ohne Hinweise auf eine Kompression in der Orbitaspitze („apical crowding“) wurden entweder durch DLWR oder pterionale Dekompression behandelt. Die Entscheidung zur Operationstechnik richtete sich hier nach Verfügbarkeit eines erfahrenen Operateurs in der jeweiligen Klinik und der Patientenpräferenz bezüglich der Technik und der induzierten Narbe. Bei klinischen Hinweisen auf eine Sehnervbeteiligung wie relativem afferentem Pupillendefekt (RAPD), Papillenschwellung oder anders nicht erklärter Visus- oder Gesichtsfeldverschlechterung sowie bei radiologischen Hinweisen auf „apical crowding“ wurde die Indikation zur pterionalen Dekompression gestellt (Abb. 1).

## Technik der Orbitadekompression über einen modifizierten pterionalen Zugang

**Figure 2:**
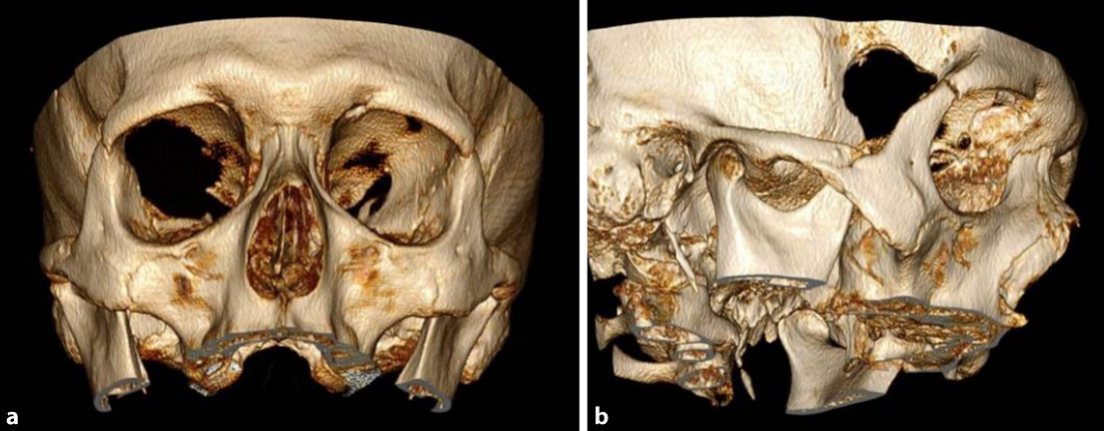

Zur Operation wird der Patient in Rückenlage positioniert. Der Kopf des Patienten wird in der Mayfield-Klemme eingespannt und dann in etwa 45° zur Gegenseite gedreht, leicht rekliniert und in dieser Position fixiert. Dann erfolgt eine minimale Rasur pterional hinter der Haaransatzlinie im Bereich der geplanten Inzision. Es wird ein kleiner bogenförmiger Hautschnitt angelegt. Nach Präparation des Temporalismuskels mit kleiner Teilinzision wird dieser unterhalb der Faszie im Bereich des frontozygomatischen Punktes abgeschoben. Mittels Rosen- und Diamantfräse werden in diesem Bereich die frontale und temporale Dura des Keilbeinsporns mit einem Durchmesser von etwa 1,5 cm dargestellt. Unter mikroskopischer Sicht werden nun entlang des Sporns und der lateralen Orbitawand sukzessive knöcherne Anteile entfernt, bis die Periorbita dargestellt ist. Anschließend werden große Teile der lateralen Orbitawand, des Orbitadaches und des Keilbeinflügels bis zur Fissura orbitalis superior entfernt. Anschließend wird die Periorbita radiär inzidiert, sodass das intraorbitale Fett- und Muskelgewebe hervortreten können. Nach einer gründlichen Blutstillung wird ein Stück Gelita eingelegt. Die Temporalismuskelfaszie wird verschlossen, und es erfolgt eine Subkutannaht, Hautnaht und -klebung. Das Operationsgebiet wird mit einem sterilen Pflasterverband gedeckt. Die Abb. 2 zeigt den postoperativen knöchernen Befund in einer 3‑D-Rekonstruktion von CT-Bildern.

## Technik der Orbitadekompression mittels lateraler Wandresektion („deep lateral wall resection“ [DLWR])

**Figure 3:**
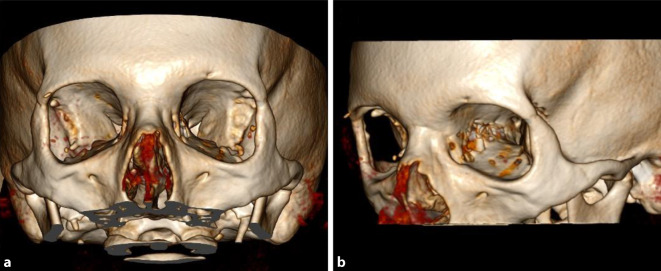

Die Entfernung der lateralen Orbitawand kann entweder über einen Swinging-eyelid-Zugang mit Durchtrennung der beiden Schenkel des lateralen Lidbändchens und Klappen des Oberlides nach oben und Unterlides nach unten erfolgen oder über einen modifizierten Blepharoplastikzugang, welcher von der Oberlidfalte ca. 2–3 cm in den vorderen Schläfenbereich ausläuft. Nach Blutstillung wird die anteriore Knochenkante der lateralen Wand dargestellt. Anschließend wird der Zugang nach unten bis zum Jochbogenansatz und nach oben bis zum Übergang in das Os frontale präpariert. Anschließend kann das Periost in der Orbitainnenseite mit einem Raspatorium abgehoben werden. Nach außen hin wird das dem lateralen Orbitapfeiler anhaftende Weichgewebe mit einem Hochfrequenzmesser oder Raspatorium bis in die Temporalisloge abgeschoben. Anschließend wird die laterale Wand von der Orbitainnenseite mit einem piezoelektrischen Schneidegerät gefenstert oder alternativ mit einem Bohrer ausgefräst. Unter sorgfältiger Visualisierung und ggf. Transillumination wird das so geschaffene Fenster immer weiter vergrößert, sodass es im unteren Bereich bis zur Fissura orbitalis inferior reicht und im oberen Bereich bis Richtung Os frontale, wobei sorgfältig darauf geachtet werden muss, nicht die vordere Schädelgrube zu eröffnen. In der Tiefe wird der sich zunehmend verdickende Knochen, welcher in das Trigonum übergeht, mit osteoklastischen Fräs- und Stanzinstrumenten so weit reduziert, dass man sich der Spitze des Temporallappens annähert, welcher meist ca. 25 mm posterior der Orbitakante liegt. Wenn der Knochen entsprechend entfernt ist, wird anschließend die Periorbita mit einem H‑förmigen Schnitt gefenstert, sodass das orbitale Fettgewebe in den neu geschaffenen Raum prolabieren kann. Erstaunlich ist dabei immer wieder, wie individuell unterschiedlich die Fließfähigkeit des orbitalen Fetts ist. Teilweise ist es gummiartig verhärtet, teilweise ist es atrophisch weich. Anschließend erfolgen eine Readaptation der subkutanen Gewebestrukturen und insbesondere der lateralen Lidaufhängestrukturen mit resorbierbarem Nahtmaterial und ein oberflächlicher Wundverschluss mit Fäden, die 10 Tage später gezogen werden. Neuronavigationssysteme präzisieren den Eingriff durch exakte anatomische Lokalisation des Situs und der Instrumente. Ebenso ist eine operative knöcherne Bildgebung mit einem C‑Bogen-CT eine gute Möglichkeit der interoperativen Qualitätskontrolle. Der postoperative knöcherne Befund ist in Abb. 3 dargestellt (3-D-Rekonstruktion einer CT nach „deep lateral wall resection“).

## Ergebnisse

### Patientenkollektiv

Das mittlere Alter des erfassten Patientenkollektivs betrug 57,6 Jahre (30 bis 84 Jahre, SD = 12,2). Der Schweregrad der Erkrankung (NOSPECS-Score) betrug im Mittel 5,6 (1–12, SD = 2,27). Nach der EUGOGO-Klassifikation wurden 43 Augen als „moderat bis schwer“ eingestuft, in 64 Fällen lag eine „visusbedrohende“ Erkrankung vor. Eine „milde“ Krankheitsschwere lag in keinem Auge vor. Die Krankheitsaktivität (CAS) betrug im Mittel 4,16 (0–14, SD = 2,42). In 57 Augen lag eine nach EUGOGO als „aktiv“ klassifizierte EO vor, in 34 Augen eine inaktive. Bei 25 Patienten (40 %) wurde Nikotinkonsum als Risikofaktor dokumentiert. Etwa ein Drittel aller Eingriffe erfolgte in Euthyreose (37/100, 37 %). Im Mittel zeigten sich erhöhte TRAK (33,0 IU/l, SD = 62) und Anti-TPO-Werte (424 U/ml, SD = 970).

Die durchschnittliche Zeitspanne zwischen der Erstdiagnose einer EO und der operativen Versorgung durch OD betrug 37 Monate (0,8–253, SD = 59). Vor dem Eingriff hatten sich 24 Patienten bereits einer Strumektomie unterzogen (38,7 %), 16 einer Radiojodtherapie (25,8 %) und 10 einer medikamentösen Thyreostase (16,1 %). Ein Patient hatte extern eine Rituximab-Gabe zur Therapie der EO erhalten (1,6 %). Die große Mehrheit der eingeschlossenen Patienten erhielt im Rahmen des klinischen Aufenthaltes eine präoperative i.v.-Steroidtherapie oder hatte eine orale oder i.v.-Steroidtherapie in der Krankengeschichte (49 Fälle, 79 %). Eine Operation wurde aufgrund persistierender Beschwerden an einem bereits erblindeten Auge durchgeführt. Die mittlere Nachbeobachtungszeit betrug 24,3 Monate (0 bis 216 Monate, SD = 43,1, 1 Monat = 30 Tage) (Tab. 1).

**Table 1:** 

*Demografie*	
Patienten	62, weiblich: 58 %, männlich: 42 %
Alter	Mittel: 57,6 Jahre (30–84, SD = 12,2)
Raucher	25 (40 %)
*Erfasste Operationen*	
Anzahl Orbitae	100
Zeitraum	2000–2019
Follow-up	Mittel: 24,3 Monate (0–216, SD = 43,1)
Indikation zur Operation	Dysthyreote Optikusneuropathie: 62 Andere: 38
Operationstechnik	Pterionale Dekompression: 83 Laterale Wandresektion: 17
*Vorbehandlungen*	
Steroidtherapie	49 (79 %)
Strumektomie	24 (39 %)
Radiojodtherapie	16 (26 %)
Thyreostase	10 (16 %)
Rituximab	1 (1,6 %)
*Krankheitsschwere zum Operationszeitpunkt*	
EUGOGO	Moderat bis schwer: 36 Visusbedrohend: 64
NOSPECS	Mittel: 5,6 (1–12, SD = 2,27)
*Krankheitsaktivität zum Operationszeitpunkt*	
Aktivität (EUGOGO)	Aktiv: 57 Inaktiv: 34 Unbekannt: 9
Aktivität (CAS)	Mittel: 4,16 (0–14, SD = 2,42)
*Krankheitsdauer zum Operationszeitpunkt*	
Monate seit Erstdiagnose EO	Mittel: 36,9 (0,8–253, SD = 59)
Operation innerhalb 24 Monate nach Erstdiagnose	Ja: 34 Nein: 13 Unbekannt: 53
*Hornhautbefund zum Operationszeitpunkt*	
Keratitis superficialis punctata	39 (39 %)
Ulkus	2 (2 %)
*Endokrine Parameter zum Operationszeitpunkt*	
Euthyreose	Ja: 37 (37 %) Nein: 42 (42 %) Keine Angabe: 21 (21 %)
TSH [µU/ml]	Mittel: 2,75 (0–33, SD = 6,9)
ft3 [pmol/l]	Mittel: 4,96 (0,92–21,3, SD = 3,5)
ft4 [pmol/l]	Mittel: 23,8 (1,1–209, SD = 34)
TRAK [IU/l]	Mittel: 33,0 (0–400, SD = 62)
Anti-TPO [U/ml]	Mittel: 424 (0–3845, SD = 970)

## Exophthalmusreduktion und Visusverbesserung

Bei den statistischen Prüfungen zeigten sich keine nennenswerten Unterschiede zwischen den Ergebnissen des t‑Tests und des *Multilevel Models* unter Berücksichtigung der Organpaarigkeit. Die nachfolgend angegebenen Werte entsprechen der Analyse durch den t‑Test. Die vollständigen Ergebnisse der statistischen Auswertung sind in Tab. 2 zusammengefasst.

**Table 2:** 

	Präoperativ	Postoperativ	Verlauf^b^
*Visus am operierten Auge nach Schweregrad*			
Visusbedrohend (*n* = 64)	0,33 (0–1,25)	0,57 (0,03–1,25)	+2,2 Zeilen (*p* < 0,001)
Nicht visusbedrohend (*n* = 36)	0,83 (0,4–1,25)	0,78 (0,2–1,0)	−0,2 Zeilen (n. s.)
Gesamt	0,46 (0–1,25)	0,63 (0–1,25)	1,4 Zeilen (*p* < 0,01)
*Exophthalmus am operierten Auge*	23,6 ± 3,2 mm (16,5–32 mm)	20,5 ± 3,2 mm (13–30,5 mm)	−2,9 ± 2,17 mm (−10–3 mm)
*Visus am Partneraugea nach Schweregrad*			
Visusbedrohend	0,5 (0,015–1,25)	0,66 (0,05–1,25)	+0,3 Zeilen (n. s.)
Nicht visusbedrohend	0,85 (0,4–1,25)	0,87 (0,4–1,25)	+0,1 Zeile (n. s.)
*Exophthalmus am Partnerauge*^a^	22,6 ± 3,68 mm (17–32 mm)	21,9 ± 4 mm (11–32 mm)	−0,9 mm (*p* < 0,05)
*Relativer Exophthalmusverlauf*^a^			
Präoperative Exophthalmusdifferenz	1,24 ± 1,93 mm (−2–7 mm)		
Postoperative Exophthalmusdifferenz	−0,92 ± 3,4 mm (−9–10,5 mm)		
Relative Exophthalmusreduktion^b^	2,16 mm (*p* < 0,001)		
*Doppelbilder*			
Dokumentierte Doppelbilder im Gebrauchsblickfeld, präoperativ	55 (35 Patienten)		
Dokumentierte Doppelbilder im Gebrauchsblickfeld, postoperativ	40 (27 Patienten)		
Dokumentierter, vollständiger Rückgang der Doppelbilder im Gebrauchsblickfeld nach Operation	9 (8 Patienten)		
Neu aufgetretene Doppelbilder im Gebrauchsblickfeld nach Operation	2 (2 Patienten)		
Persistierende Doppelbilder im Gebrauchsblickfeld nach Operation	37 (27 Patienten)		
*Milde Komplikationen*			
Kauschmerz	0		
Kopfschmerz	3		
Sensibilitätsstörung	4		
Relevante Nachblutung	2		
Stirnastparese des N. facialis	1		
Fälle mit milden Komplikationen (gesamt)	9		
*Schwere Komplikationen*			
Neue Doppelbilder im Gebrauchsblickfeld	2		
Visusminderung >3 Zeilen	1		
V. a. Orbitaphlegmone	1		
Tränendrüsenschädigung	1		
Fälle mit schweren Komplikationen (gesamt)	4		
*Dauer des stationären Aufenthaltes*	Mittel: 8 Tage (2 bis 24 Tage, SD = 4,35)		

^a^Die Berechnung dieser Werte erfolgte nur für den ersten Eingriff pro Patient (*n* = 62). Alle postoperativen Werte wurden vor einer etwaigen Operation des FE erhoben

^b^Berechnet als Mittelwert der individuellen Differenzen

**Figure 4:**
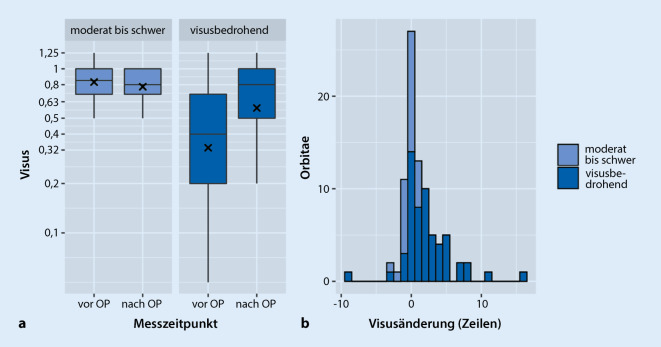

**Figure 5:**
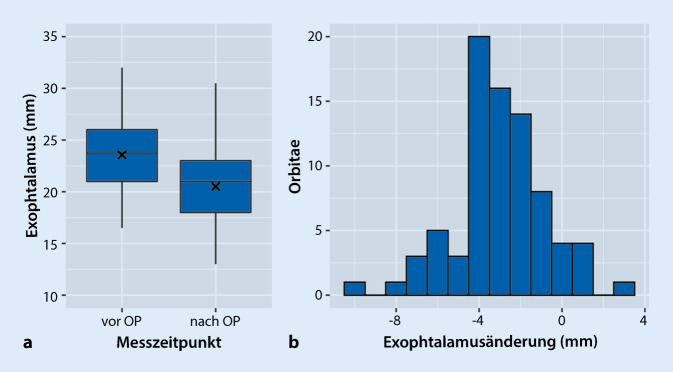

Das Sehvermögen von Augen mit visusbedrohendem Schweregrad verbesserte im Mittel um 2,2 Zeilen von 0,33 auf 0,57 (*p* < 0,001). Operationen aus rehabilitativer Indikation beeinflussten das Sehvermögen kaum (mittlerer Visus präoperativ 0,83, postoperativ 0,78, *p* = n. s.) (Abb. 4). Der mittlere Visusgewinn aller Augen betrug 1,4 Zeilen (*p* < 0,001). Die mittlere postoperative Exophthalmusreduktion im gesamten untersuchten Kollektiv betrug 2,9 mm (95 %-CI: 2,45–3,42 mm, *p* < 0,01) (Abb. 5). Im Vergleich zum Partnerauge zeigte sich eine relative Reduktion von 2,16 mm (95 % CI: 0,98–3,34).

Der Visus des Partnerauges (FE) blieb im Beobachtungsintervall bei beiden Schweregraden stabil, jedoch zeigte sich eine leichte, statistisch signifikante Reduktion des Exophthalmus um 0,9 mm von 22,6 mm präoperativ (17–32 mm, SD = 3,68) auf 21,9 mm nach der Operation (11–32 mm, SD = 3,99 mm).

## Doppelbilder

In dem vorliegenden Kollektiv zeigte die Intervention wenig Einfluss auf die klinische Angabe von Doppelbildern im Gebrauchsblickfeld: Präoperativ waren bei 55/100 Operationen (35 Patienten) beklagte Doppelbilder dokumentiert, postoperativ nach 40 Eingriffen (27 Patienten) Bei 2 Eingriffen an 2 Patienten ohne präoperativ beklagt Doppelbilder wurden postoperativ neue Doppelbilder dokumentiert (3,2 % aller Patienten). Nach 9 Eingriffen an 8 Patienten wurde ein vollständiger Rückgang präoperativ beklagter Doppelbilder dokumentiert (12,9 % aller Patienten).

## Komplikationen

Nach den meisten Eingriffen wurden postoperativ keine oder nur milde Komplikationen dokumentiert. Milde Komplikationen traten nach 9 von 100 Operationen auf (9 %). Hiervon wurde ein postoperativer Kopfschmerz in 3 Fällen dokumentiert. Bei 1 Patientin bestand postoperativ der Verdacht auf eine retrobulbäre Nachblutung. Der Augeninnendruck war auf 26 mm Hg erhöht, durch Kühlung konnte eine zögerliche Befundbesserung mit Normalisierung des Augeninnendruckes erreicht werden. Ein weiteres postoperatives, periokuläres Hämatom war ohne Intervention spontan regredient, der Visus lag 6 Monate nach der Operation bei 1,0. Eine Sensibilitätsstörung wurde nach 4 Eingriffen dokumentiert. In einem Fall kam es zu einer reversiblen Stirnastparese des N. facialis. Schwere Komplikationen wurden nach 4 von 100 Eingriffen beschrieben (4 %): In 2 Fällen kam es nach pterionaler Dekompression zu neuen postoperativen Doppelbildern. Ein Auge mit dysthyreoter Optikusneuropathie erfuhr wenige Tage nach pterionaler Dekompression einen Visusabfall von 0,8 auf 0,1. Besondere Risikofaktoren lagen nicht vor. Eine postoperative Bildgebung zeigte keine Hinweise auf eine Schädigung des N. opticus, der Grund für die Sehminderung konnte nicht sicher bestimmt werden. Die Tränendrüse einer Patientin wurde nach pterionaler Dekompression geschädigt, was eine dauerhafte Benetzungstherapie nötig machte. Der Visus war stabil bei 0,7. Ein Patient wurde postoperativ nach DLWR bei Verdacht auf Orbitaphlegmone antibiotisch therapiert, worunter sich rasch eine Befundbesserung zeigte. In weiteren Nachuntersuchungen über 2 Jahre zeigten sich keine bleibenden Beeinträchtigungen, der Visus betrug 1,0.

## Diskussion

Die chirurgische Orbitadekompression (OD) ist seit der Erstbeschreibung des pterionalen Zugangs durch Krönlein 1888 [21] und Modifikation durch Dollinger 1911 [22] fester Bestandteil der Orbitachirurgie und der Therapie der EO [23]. Das chirurgische Repertoire an Zugängen und Abtragungsorten wurde seither stetig verbreitert, was zu einer Verbesserung der Nutzen-Risiko-Abschätzung führte und eine breitere Indikationsstellung ermöglichte [14]. Es handelt sich jedoch weiterhin um einen vergleichsweise seltenen Eingriff, der hauptsächlich in spezialisierten Zentren durchgeführt wird. So berichteten in einer Umfrage der *American Society of Ophthalmic Plastic and Reconstructive Surgery* 45 %, weniger als 10 Dekompressionen/Jahr, und 30 %, weniger als 20 Dekompressionen/Jahr durchzuführen. Die Mehrheit war seit über 15 Jahren operativ tätig [24].

Ein wesentlicher Aspekt bei der Weiterentwicklung der operativen Verfahren ist die Vermeidung induzierter Doppelbilder. Diese traten bei der in der frühen zweiten Hälfte des 20. Jahrhunderts oft durchgeführten transantralen Abtragung der medioinferioren Wand in ca. 50–68 % der Fälle auf [25–28]. Durch den von Kennedy 1990 beschriebenen transnasal-endoskopischen Zugang konnte die Häufigkeit neuer Doppelbilder reduziert werden, Angaben zur Inzidenz schwanken jedoch stark [29–33]. Eine 2015 veröffentlichte Querschnittstudie mit längerer Nachbeobachtungszeit berichtete von neuer Diplopie in 19 % und einer Verschlechterung in 22 % [34]. Die mittlere Exophthalmusreduktion bei diesem Zugang liegt bei ca. 3,5 mm bei ausschließlicher Dekompression der medialen Wand.

Es ist intuitiv, dass durch Entfernung medialer Knochenanteile v. a. eine Esotropie induziert wird, durch Entfernung lateraler Anteile eher eine Exotropie. Dies wurde durch mehrere Beobachtungen bestätigt [35, 36]. Leone et al. beschrieben 1989 eine „balancierte“ Dekompressionsmethode mit Entfernung von medialen und lateralen Wandanteilen mit der Intention, die Rate an neuen Doppelbildern zu verringern [37]. In einer kürzlich publizierten, großen Fallserie (*n* = 318 Orbitae) wurden neue Doppelbilder in 22–26 % gefunden bei einer Exophthalmusreduktion von 4,6–5,3 mm. Weitere Studien berichten eine Rate an iatrogener Diplopie von 0–53 % und eine Exophthalmusreduktion von 2,5–6 mm [15, 36, 38–40].

Die laterale Wand, deren Abtragung bereits 1911 durch Dollinger erstbeschrieben wurde [22], rückte zuletzt wieder vermehrt in den Fokus der Orbitachirurgen [15]. Der Zugang ist beispielsweise durch Oberlidfaltenschnitt, Swinging-eyelid-Zugang, transkonjunktival oder auch transantral möglich. Als Erweiterung der lateralen Orbitadekompression wurde die „deep lateral wall resection“ (DLWR) von Goldberg et al. beschrieben. Die Autoren identifizierten 3 tief gelegene, knöcherner Bereiche („Türpfosten“ der Ala major ossis sphenoidalis, lakrimales „Schlüsselloch“ und „Becken“ der Fissura orbitalis inferior) der lateralen Wand mit insgesamt durchschnittlich 5,6 cm^3^ potenziellem Resektionsvolumen [20]. Mehrere vergleichende Arbeiten bescheinigen der DLWR eine geringere Rate an induzierter Diplopie als der balancierten Dekompression [41]. So fanden Rocchi et al. eine Inzidenz von neuen Doppelbildern im Geradeausblick von 0 % nach lateraler und 17,8 % nach balancierter OD bei einer mittleren ER von 4 mm bzw. 5,7 mm. [42]. Baldeschi et al. kamen in einer vergleichenden Studie zu dem Schluss, dass eine zusätzliche Abtragung der tiefen lateralen Knochenanteile im Rahmen einer 3‑Wand-Dekompression eine zusätzliche ER von 2,3 mm erbrachte, ohne das Risiko postoperativer Diplopie zu erhöhen [43]. Der Chirurg kann hier demnach oberflächlich beginnen und die knöcherne Resektion je nach Bedarf auf die von Goldberg et al. beschriebenen tiefen Teile ausdehnen. In einer der wenigen prospektiven und randomisierten Studien in dem Feld von Franceschini et al. mit 38 Teilnehmern (76 Orbitae) zeigte sich jedoch kein Vorteil der lateralen gegenüber der balancierten Dekompression. Bei beiden Eingriffen trat keine neue Diplopie auf. Bei Patienten mit präoperativ vorhandenen Doppelbildern traten bei beiden Techniken etwa gleich häufig (40 %) neue Doppelbilder im Geradeausblick auf [44]. Unsere Arbeit bestätigt die guten Erfahrungen bei DLWR bezüglich neuer Diplopie, jedoch ohne einen Vergleich zur balancierten Dekompression.

Ebenfalls effektiv zur Vermeidung postoperativer Diplopie ist die pterionale Dekompression, die jedoch vergleichsweise selten eingesetzt wird. Eine Fallserie von Algvere et al. (*n* = 22 Orbitae) berichtet eine durchschnittliche Exophthalmusreduktion von 4–5,7 mm ohne neue postoperative Diplopie. Bei einem Patienten mit präoperativ blasser Papille kam es im Verlauf von 3 Jahren zu einer Optikusatrophie [45, 46]. Schick und Hassler erreichten eine Verringerung des Exophthalmus um 4,8 mm bei 20 Augen, ebenfalls ohne neu induzierte Doppelbilder. Ein Patient musste aufgrund eines Epiduralhämatoms revidiert werden, zeigte jedoch keine weiteren Folgen [47]. Die größte Serie stammt von Korinth et al. (*n* = 59 Orbitae) mit einer Exophthalmusreduktion von 3,8 mm, ebenfalls ohne berichtete neue Diplopie. Die wichtigsten Komplikationen waren eine permanente Fazialisparese in 2 Fällen und 1 Fall von „reversiblem schmerzhaftem Exophthalmus“ [17]. In unserem Kollektiv kam es in 1 Fall mit DON nach pterionaler Dekompression zu einer postoperativen Sehminderung auf 0,1, vermutlich aufgrund einer mikrovaskulären Ischämie. In der Literatur sind mehrere Fälle mit Sehminderung nach Orbitadekompression beschrieben, beispielsweise durch Nishumura et al. nach transkarunkulärem Eingriff [48]. In einer Studie der *British Ophthalmological Surveillance Unit* (BOSU) wurden innerhalb eines Jahres 71 Augen mit DON in Großbritannien erfasst, von denen eines durch eine intraoperative Blutung eine Visusminderung auf LSP erlitt [49]. Patienten sollten auf die Möglichkeit dieser seltenen, aber schweren Komplikation hingewiesen werden.

Ein weiterer Ansatz zur Versorgungsoptimierung besteht in einer patientenorientierten Wahl des Operationsverfahrens [15]. So berichteten McCann et al. von Erfahrungen mit medialer transkarunkulärer Orbitadekompression bei DON, jedoch DLRW mit Fettresektion bei rehabilitativer Indikation [18]. Sie erreichten durch dieses Behandlungsschema eine Visusverbesserung von 0,13 auf 0,66 bei DON, die Exophthalmusreduktion durch den lateralen Eingriff betrug 2,6 mm bei 2,6 % induzierter neuer Diplopie im Geradeausblick [16]. In Analogie hierzu wählten wir in unserem Patientenkollektiv für Patienten mit DON oder Anzeichen von „apical crowding“ eine pterionale Dekompression. Patienten mit rehabilitativer Indikation wurden mit pterionaler oder tiefer lateraler OD behandelt. Durch diesen Entscheidungsprozess erreichten wir in unserem Kollektiv ähnliche Ergebnisse mit einer suffizienten Exophthalmusreduktion (2,9 mm), Visusrehabilitation bei bedrohtem Sehvermögen und einer neuen Diplopie im Gebrauchsblickfeld bei 2 Patienten. Der von uns beobachtete mittlere Visusanstieg von 1 bis 2 Zeilen über das gesamte Kollektiv entspricht dem von Leong et al. errechneten Mittelwert in der untersuchten Literatur [12]. Der Anteil der Patienten mit Dysthyreose und aktiver Erkrankung ist v. a. durch die hohe Zahl der aufgrund einer DON dekomprimierten Fälle zu erklären. Auch 10 Patienten mit rehabilitativer Indikation waren zum Operationszeitpunkt nach Aktenlage hyperthyreot, diese waren jedoch bereits thyreodektomiert oder strahlentherapiert. Dies könnte durch eine Übersubstitution mit Schilddrüsenmedikamenten (Hyperthyreosis factitia) bedingt sein.

Da wir in dieser Studie die Visus- und Exophthalmuswerte sowohl des operierten Auges als auch des Partnerauges erhoben, konnten wir die Veränderung dieser Werte zwischen dem ersten operierten Auge und dem Partnerauge vergleichen. Das Sehvermögen des Partnerauges blieb konstant, jedoch zeigte sich eine leichte (−0,9 mm), statistisch signifikante Abnahme des Exophthalmus. Dieser Effekt ist entweder durch ein Nachwirken einer präoperativen Steroidtherapie oder den natürlichen Krankheitsverlauf bedingt und kann zu einem Überschätzen der operationsbedingten Verbesserung führen.

Unsere Studie ist limitiert durch ihren retrospektiven Charakter. So erlaubte die Aktenlage lediglich suffiziente Aussagen zur Angabe von Doppelbildern im Gebrauchsblickfeld. Präzise Schlussfolgerungen zum zeitlichen Verlauf aller Parameter sind aufgrund der nicht standardisierten Nachbeobachtung nicht möglich [12].

## Schlussfolgerung

Unsere Studie bestätigt die Rolle der Orbitadekompression bei visusbedrohender und schwer beeinträchtigender endokriner Orbitopathie. Die große Mehrheit der behandelten Patienten kann mit einer postoperativen funktionellen Verbesserung von Visus und Exophthalmus rechnen. Die Komplikationsrate ist insgesamt gering, aber Patienten müssen über die Möglichkeit schwerwiegender Ereignisse wie einer Visusminderung oder das Auftreten postoperativer Diplopie aufgeklärt werden. Zukünftige Studien sollten auf eine größtmögliche Vergleichbarkeit der erhobenen Daten bedacht sein. Präoperative Messwerte sollten unmittelbar vor dem Eingriff und nach erfolgter konservativer Therapie erhoben werden. Eine Erhebung aller Zielparameter auch für das nicht operierte Auge kann helfen, eine Attribution systemischer Effekte an den operativen Eingriff zu vermeiden.

## Footnotes

1 Das englische Akronym „NOSPECS“ bezieht sich auf die von Werner et al. 1969 vorgeschlagene und später revidierte Einteilung [5, 6]: ***N****o signs or symptoms* (Klasse 0), ***O****nly signs, no symptoms* (Klasse 1), ***S****oft tissue involvement* (Klasse 2), ***P****roptosis* (hier gemeint: Exophthalmus, Klasse 3), ***E****xtraocular muscle involvement* (Klasse 4), ***C****orneal involvement* (Klasse 5), ***S****ight loss* (Klasse 6). In unserer Klinik üblich ist ein Punktesystem, bei dem innerhalb verschiedener Kategorien jeweils 0 bis 3 Punkte vergeben werden.
